# Zoonotic dermatophyte *Trichophyton erinacei* in pet hedgehogs: sampling methodology and further insight into genetic diversity

**DOI:** 10.3389/fvets.2026.1755936

**Published:** 2026-07-01

**Authors:** Petr Cibulka, Vít Hubka, Adéla Wennrich, Miroslav Kolařík, David Modrý

**Affiliations:** 1 Department of Veterinary Sciences, Faculty of Agrobiology, Food and Natural Resources/CINeZ, Czech University of Life Sciences Prague, Prague, Czechia; 2 Department of Botany, Faculty of Science, Charles University, Prague, Czechia; 3 Laboratory of Fungal Genetics and Metabolism, Institute of Microbiology, Czech Academy of Sciences, Prague, Czechia; 4 Department of Botany and Zoology, Faculty of Science, Masaryk University, Brno, Czechia; 5 Institute of Parasitology, Biology Center of Czech Academy of Sciences, Ceské Budějovice, Czechia

**Keywords:** African pygmy hedgehog, *Atelerix*, dermatophytosis, emerging disease, zoonosis

## Abstract

*Trichophyton erinace*i is a zoonotic dermatophyte, typically associated with hedgehogs. While the European hedgehog (*Erinaceus europaeus*) is a frequent host in the wild, the African pygmy hedgehog (*Atelerix albiventris*) is increasingly affected in captive settings. Despite carrying the fungus, most hedgehogs remain asymptomatic, which complicates early detection and increases the risk of transmission to other animals or humans. This study addresses the difficulty of sampling from asymptomatic individuals and the need for understanding the genetic variation of *T. erinacei*. Three non-invasive sampling techniques (interdental brushes, cotton swabs, and toothbrushes) were used to collect skin samples from 103 captive *A. albiventris* individuals in the Czech Republic and Romania. Other materials, such as spines or crusts, were also collected. Samples were cultured on selective media (SDA with cycloheximide and chloramphenicol), and isolates were identified by ITS rDNA region sequencing. Genetic variation was assessed using multilocus microsatellite typing. Out of 103 sampled African pygmy hedgehogs, 24.3% (25/103) tested positive for *T. erinacei*, of which 76.0% (19/25) were asymptomatic. No other dermatophyte species were isolated from tested hedgehogs. Among different sampling techniques, interdental brushes showed the highest detection rate (80%), significantly outperforming cotton swabs and toothbrushes. ITS rDNA region sequencing and multilocus microsatellite typing revealed no genetic variation among the isolates, indicating low intraspecific diversity in *T. erinacei* from captive *A. albiventris*. Our results emphasize the importance of reliable, non-invasive sampling methods for early diagnosis, particularly in the context of rising numbers of hedgehogs kept as pets. It also demonstrates the superior effectiveness of interdental brushes over other sampling techniques for detecting *T. erinacei* in hedgehogs, offering a practical improvement for pathogen surveillance. The absence of genetic variability suggests the circulation of a single clone M1 in the captive population.

## Introduction

*Trichophyton erinacei* (*Onygenales, Arthrodermataceae*) is a zoophilic dermatophyte that infects mammals, especially hedgehogs (1, 2, 52). It is one of the zoonotic dermatophytes increasingly reported as being acquired from pet and exotic animals in Western Europe and some Asian countries (1–6). The fungus probably co-evolved with hedgehogs as its main hosts and spread beyond its original range through biological invasions (7) and the pet trade (1, 3, 8).

While the hedgehog family Erinaceidae comprises 19 species of Old-World insectivores (53), *T. erinacei* has been confirmed in only three: *Erinaceus europaeus, Atelerix albiventris*, and *Hemiechinus auritus* (1, 2, 5–7). The occurrence in *Erinaceus roumanicus* is questionable due to the limited and possibly inaccurate data of identification of the hedgehog species (9, 10). A recent study from Romania showed that the *E. roumanicus* carry a dermatophyte from the genus *Trichophyton*, but the study focused only on morphology and no species determination was performed at the molecular level (11).

As hedgehogs, especially *A. albiventris* (the White-bellied hedgehog, Four-toed hedgehog or African pygmy hedgehog), become increasingly popular as pets, the risk of transmission of *T. erinacei* increases ((1, 2, 6, 12–14, 54), particularly because many owners lack awareness of zoonotic risks associated with this pet (12, 15). In humans, infections usually manifest as itchy, red, and scaly patches on the skin, most commonly on arms and hands (16–20). In contrast, infections in hedgehogs are frequently asymptomatic, but may also cause scaly skin lesions, loss of spines and alopecia in some individuals (5, 6).

Besides its direct pathogenicity, *T. erinacei* is thought to contribute to the development of antimicrobial resistance in some bacterial species, in particular *Staphylococcus aureus* and its notoriously known methicillin-resistant form MRSA (2, 7, 21, 22, 55) which has been the subject of numerous studies in recent years (21–25, 55). The association between MRSA in hedgehogs and *T. erinacei* suggests a co-evolutionary relationship (23, 26, 55) and underscores the need to understand the genetic diversity of *T. erinacei* from both the clinical and ecological perspectives.

*Trichophyton erinacei* is a member of the *Trichophyton benhamiae* complex, together with several other zoophilic species, such as *T. benhamiae, T. bullosum, T. europaeum, T. verrucosum, T. eriotrephon* and others (18, 27, 28). Molecular techniques such ITS (Internal Transcribed Spacer) rDNA region sequencing, multilocus microsatellite typing (MLMT) and multilocus sequence typing (MLST) (2) were used to assess genetic variability among *T. erinacei* isolates. Two main subpopulations of *T. erinacei* have been identified: one associated with pet African hedgehogs and the second, consisting mainly of the isolates from wild European hedgehogs (*E. europaeus*) (2, 29, 30). Although microsatellite markers, multi-locus sequence data, conidial dimensions, and antifungal susceptibility profiles revealed certain differences between these two populations, these variations were not considered sufficient to justify taxonomic separation, and *T. erinacei* was therefore treated a genetically and phenotypically variable yet taxonomically unified species (2).

Common methods used in small animal dermatology for the diagnosis of dermatophytosis are usually non-invasive or semi-invasive, however they are usually based on scraping the affected areas or skin collection of scabs and crusts and removing spines with a bulb (17, 31). Some of these methods for hedgehog sampling can only be performed in the veterinary clinic by trained professionals on properly immobilized or anesthetized animals because of the level of stress and welfare of the animal. The swab method used in diagnostics of dermatophytosis employs simple sampling tools such as toothbrushes, sterile cotton swabs, hairbrushes and small carpet squares (5, 31–33, 56). These tools are well suited for collecting samples from both symptomatic individuals with skin or hair lesions and asymptomatic carriers and are applicable in both clinical and field settings (31–34). However, this approach is often neglected in preventive diagnostics and in the screening of clinically healthy individuals.

The aims of this study are (i) to optimize sampling methodology for the cultivation of dermatophytes from asymptomatic pet hedgehogs, and (ii) to gain insight into the ongoing spread and genetic diversity within *T. erinacei* populations in captive hedgehogs in the Czech Republic and Romania.

## 2 Materials and methods

### 2.1 Material origin

The samples (*n* = 309) were collected from 103 African pygmy hedgehogs between February and December 2023. For each hedgehog, three samples were obtained using three different techniques (interdental brush, cotton swab and toothbrush). The study was conducted in collaboration with private owners from the Czech Republic (Prague, Chomutov, Police nad Metují, Žalhostice), and two veterinary clinics specializing in exotic pets: VetExotic (Prague, Czech Republic) and Veterinary Clinic Serviciul Noi Animale de Companie USAMV (Cluj-Napoca, Romania). Information was recorded on the location, sex, age, weight, origin of the animals and clinical signs of dermatophytosis.

### 2.2 Sampling

Three types of sampling tools were used for each individual: (i) interdental brush (TePe^®^, 1.5 mm; 3,795), (ii) cotton swab (Linteo^®^ 1001/2) and (iii) Select MEDIUM toothbrush (TePe^®^ 24030). Tested techniques were adapted from the brushing techniques described by Mackenzie (35) and Moriello (31). Prior to sampling, hedgehogs were examined for the presence of visible skin lesions. Samples were collected using a single tool across multiple body regions, including the dorsal surface between the spines, the abdomen, the head, and the lateral transition zone between the spines and hairs, as well as from lesion sites when present. All three sampling tools were used in parallel. Each sample was sealed in a separate zip-lock bag. In cases of dry, flaky skin or broken spines, these parts were removed and stored in separate zip-top bags, while scabs were carefully scraped off using tweezers and a scalpel and stored in the same manner, but only with the owner's consent. All samples were stored in a refrigerator at 3 °C−7 °C.

### 2.3 Cultivation

The sample was plated on Sabouraud dextrose agar (SDA) containing cycloheximide and chloramphenicol (Trios, Prague, Czech Republic; MKM 03019). The plates were incubated at 25 °C for 14 days, with checks every 2–3 days. Additional material, like spines, hairs and crusts were plated on separate plates by pressing the material directly into the agar, cultivated under the same conditions and examined in the same way. Colonies showing morphology characteristics of dermatophytes were subjected to microscopic examination and molecular identification by sequencing the internal transcribed spacer (ITS) region of ribosomal DNA. Selected isolates were deposited into the Culture Collection of Fungi (CCF), Department of Botany, Charles University, Prague, Czech Republic.

### 2.4 DNA extraction and PCR

DNA was extracted from colonies grown on malt extract agar (MEA; malt extract from Oxoid, Basingstoke, UK) and incubated at 25 °C for 10 days. A commercial kit ZR Fungal/ Bacterial DNA Kit™ (ZYMO RESEARCH, Irvine, California, USA) was used and the instructions from the manual were followed. The PCR reaction volume of 20 μl contained 1 μl (50 ng ml^−1^) of DNA, 0.3 μl of both primers (25 pM ml^−1^), 0.2 μl of MyTaqTM DNA Polymerase (Bioline, GmbH, Germany) and 4 μl of 5 × MyTaq PCR buffer. The quality of the DNA was verified using the NanoDrop 2000 Spectrophotometer (Thermo Fisher Scientific, USA). The ITS and partial LSU (Large Subunit) rDNA region was amplified using primers ITS1F (CTTGGTCATTTAGAGGAAGTAA) and NL4 (GGTCCGTGTTTCAAGACGG), following the protocol described by Hubka et al. (36). PCR amplicons were purified with EXOSAP PP-218L (Jena Bioscience, Jena, Germany) and sequenced using both terminal primers by Sanger sequencing Bioanalyzer 2100 (Agilent, Santa Clara, USA) at BIOCEV, Czech Republic. The sequences were inspected and assembled using Bioedit v. 7.2.5 (https://bioedit.software.informer.com/2/). Identification was performed by comparing the ITS sequences obtained with sequences derived from ex-type strains of the *T. benhamiae* species complex (2, 28).

### 2.5 Phylogenetic analyses

ITS rDNA sequences obtained in this study were combined with the dataset published by Cmoková et al. (2). Alignment of the ITS rDNA region was performed using the FFT-NS-i (Fast Fourier Transform-Normalized Similarity-iterative) option implemented in the MAFFT (Multiple Alignment using Fast Fourier Transform) online service (37). The alignment was trimmed, concatenated and then analyzed using the maximum likelihood (ML) method. The final alignment contained 592 positions, of which 88 were variable and 53 were parsimony—informative. Suitable partitioning schemes and substitution models were selected based on the Bayesian Information Criterion (BIC), allowing different segments of the ITS region to be treated as independent datasets. The optimal partitioning scheme was as follows: ITS1 and ITS2 (TrN+G); 5.8S (K80). The ML tree was constructed using IQ-TREE v. 1.4.4 (38), with nodal support determined by ultrafast bootstrap (UFBoot) with 100,000 replicates. The tree was rooted using *Trichophyton rubrum*.

### 2.6 Microsatelites MLMT

Microsatellite-based subtyping of *T. erinacei* was conducted following the typing protocol previously developed for members of the *T. benhamiae* clade (28). PCR amplifications were set up in a final volume of 5 μl, containing approximately 50 ng of template DNA, 0.5 μl of a mixed primer solution, and 2.5 μl of Multiplex PCR Master Mix (Qiagen, Germany). Thermal cycling parameters were applied in accordance with the manufacturer's guidelines. Amplification products were diluted 25-fold with water, then combined with 10 μl of deionized formamide and 0.2 μl of GeneScan™ 600 LIZ size standard. The mixture was denatured at 95 °C for 5 min and analyzed using an ABI 3100 Avant Genetic Analyzer. The allele dataset was produced using GeneMarker v. 1.51 (SoftGenetics, LLC, State College, PA, USA) and subsequently compared to the dataset and multilocus genotypes M1-M10 reported by Cmoková et al. (2). All positive samples were examined by MLMT.

### 2.7 Statistical analysis

A positive sample reference standard was defined as a sample detected by at least one of the three tested techniques, or any other sample, such as skin, spines or crusts, taken from tested animal. The comparison of the sampling techniques with real positive samples was analyzed using the statistical software TIBCO Statistica (14.00.00) with a Chi-square test and Cochran's *Q* test for comparison between each sampling techniques. The significance level was set at α = 0.05.

## 3 Results

### 3.1 Characteristics of the examined hedgehog population

Out of 103 sampled hedgehogs, 100 individuals (97%) originated from private Czech breeders, while 3 hedgehogs came from Romanian pet shops. The sex ratio was nearly balances, with 51 males and 52 females. The average weight was 366.7 grams (range: 150–650 g). The average age was 13.9 months (range: 4–60 months). Skin lesions were observed in 13 animals (12.6%).

### 3.2 Prevalence

**Figure 1:**
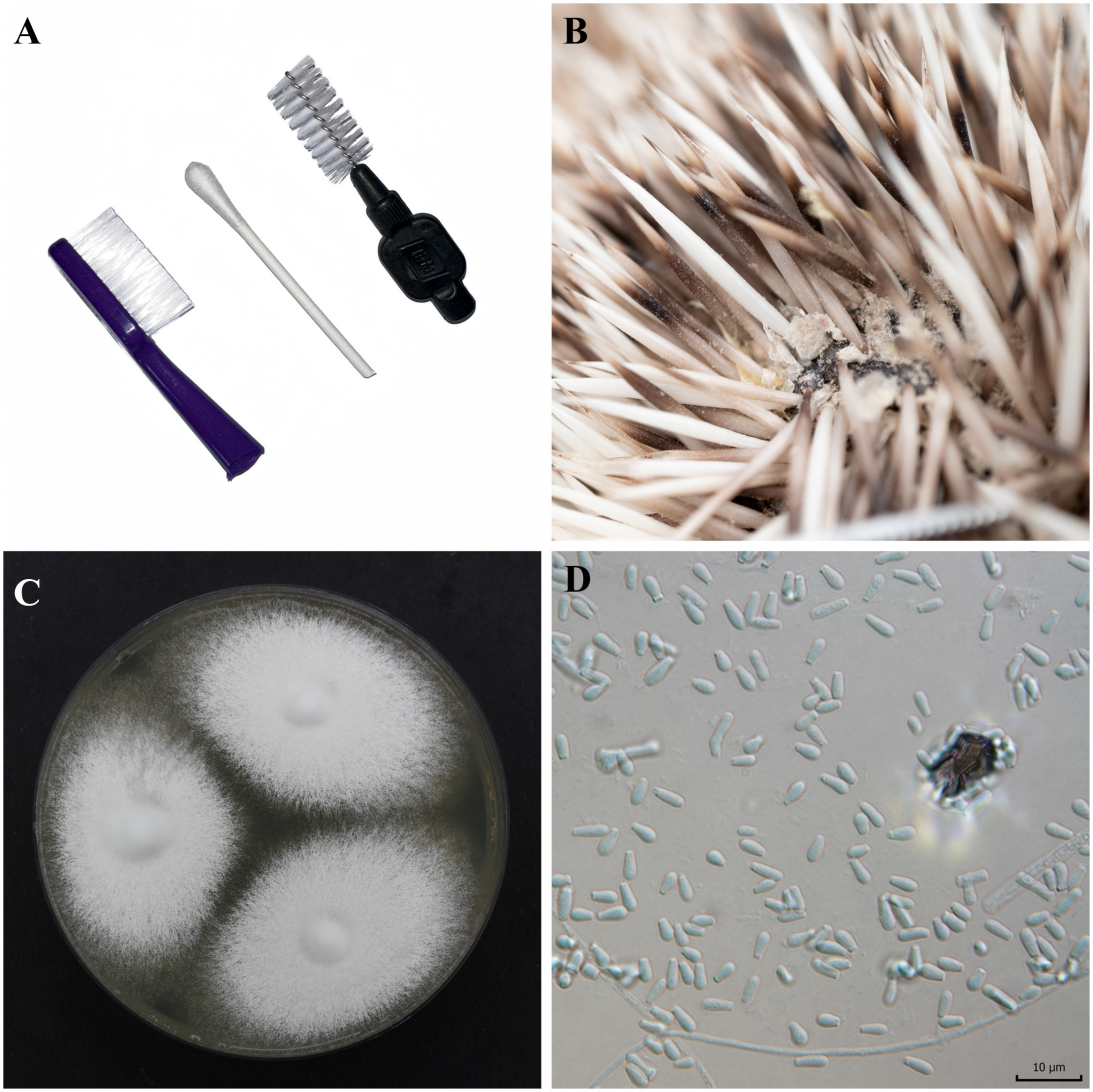
Sampling procedures and morphology of *T. erinacei*. **(A)** Sampling tools utilized for fungal collection (toothbrush, cotton swab, and interdental brush). **(B)** Clinical presentation showing scaly skin and hyperkeratosis at the base of the quills on a symptomatic hedgehog. **(C)** White, fluffy colonial morphology of *T. erinacei* after 14 days of incubation. **(D)** Microscopic examination revealing numerous characteristic microconidia (lactophenol cotton blue, magnification 100x).

Of the total number of cultured isolates, 25 were found to be positive for dermatophyte species (24.3%), as determined by their macroscopic and microscopic characteristics on selective medium (Figure 1). *T. erinacei* was the only fungal species isolated and identified by ITS sequencing. Of the 25 positive animals, six (24%) were females, and 19 (76%) males.

### 3.3 Asymptomatic carriers

The majority of infected hedgehogs were asymptomatic, with 76% (19/25) showing no visible clinical signs of dermatophytosis. Among the 13 animals with skin lesions, only six (46.2%) tested positive for *T. erinacei*.

### 3.4 Comparison of sampling techniques

Of the 25 *T. erinacei* positive animals, 20 were detected by interdental brush (80%), nine by cotton swab (36%) and nine by toothbrush (36%). Only four animals tested positive with all three techniques. The interdental brush failed to detect three positive cases, which were proven positive by cotton swab (*n* = 3) and toothbrush (*n* = 1). In two additional animals, all three swabbing methods yielded negative results, but *T. erinacei* was successfully cultured from spines or crusts. Detailed results are presented in Supplementary Table S1.

Additional material (spines, scales, or crusts) was collected from six hedgehogs with skin lesions. Cultures were positive in five animals, including two cases in which *T. erinacei* was detected exclusively from the additional material, while all corresponding swabs remained negative.

### 3.5 Statistical comparison of sampling techniques

Statistical analysis (Chi-square test) confirmed a significant association between each sampling technique and culture positivity; interdental brush (*p* = 0.025), cotton swab (*p* = 0.00006), and medium toothbrush (*p* = 0.00006) (Table 1). The test showed that the interdental brush is the most sensitive, but with a low statistically significant association between technique and outcome. Cochran's *Q* test revealed a statistically significant difference in detection efficacy among the three sampling techniques (*Q* = 31.16, *p* < 0.000001), indicating that the performance of at least one method differed significantly from the others. Subsequent McNemar Chi-square test revealed significant pairwise differences between interdental brush and both cotton swab (*p* = 0.0412), and interdental brush and toothbrush (*p* = 0.0133) while the difference between cotton swab and toothbrush was not statistically significant (*p* = 0.0736).

**Table 1:** Sensitivity of different methods of sampling. Chi-square test was used to compare three sampling techniques with a reference standard (sample detected by at least one of the three tested techniques, or any other sample, such as skin, spines or crusts, taken from tested animal). A total of 25 out of 103 animals were positive.

Method of Sampling	Number of samples	True positive	False negative	Sensitivity	p-value
Interdental brush	103	20	5	80.0 %	0.025
Cotton swab	103	9	16	36.0 %	0.00006
Toothbrush	103	9	16	36.0 %	0.00006

### 3.6 Genetic diversity of *T. erinacei*

**Figure 2:**
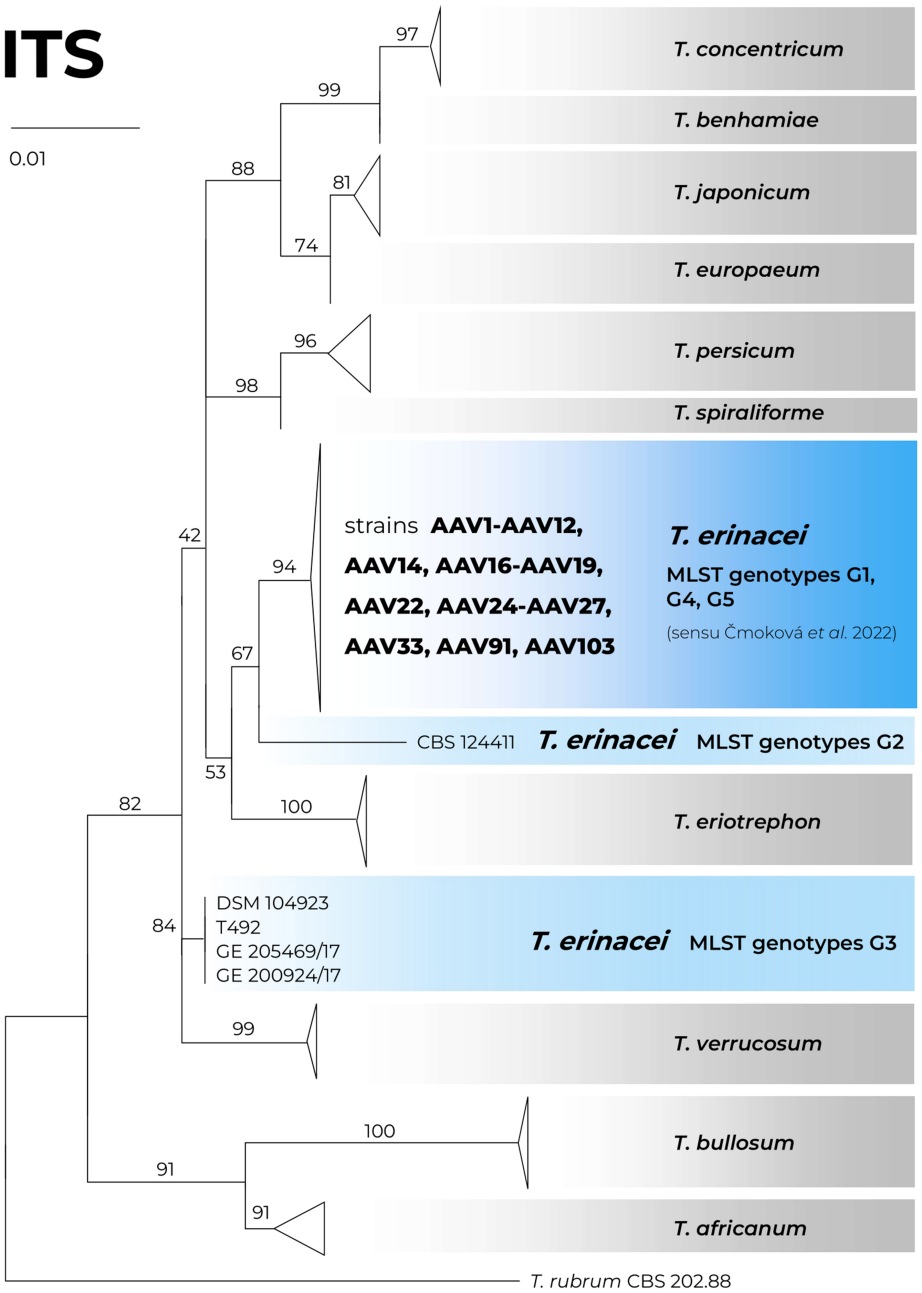
Molecular phylogenetic tree of ITS sequences of Trichophyton erinacei of pet *A. albiventris* from Czech Republic and Romania. ITS rDNA sequences obtained in this study were combined with the dataset published by Cmoková et al. (2). The strains obtained for this study are listed under the abbreviations AAV and are highlighted in bold.

Sequence analysis of the ITS and partial LSU rDNA region revealed no genetic variation among 25 *T. erinacei* isolates from *A. albiventris* isolated in the Czech Republic and Romania (Figure 2). The accession numbers for the ITS and LSU sequences are shown in Supplementary Table S1.

**Figure 3:**
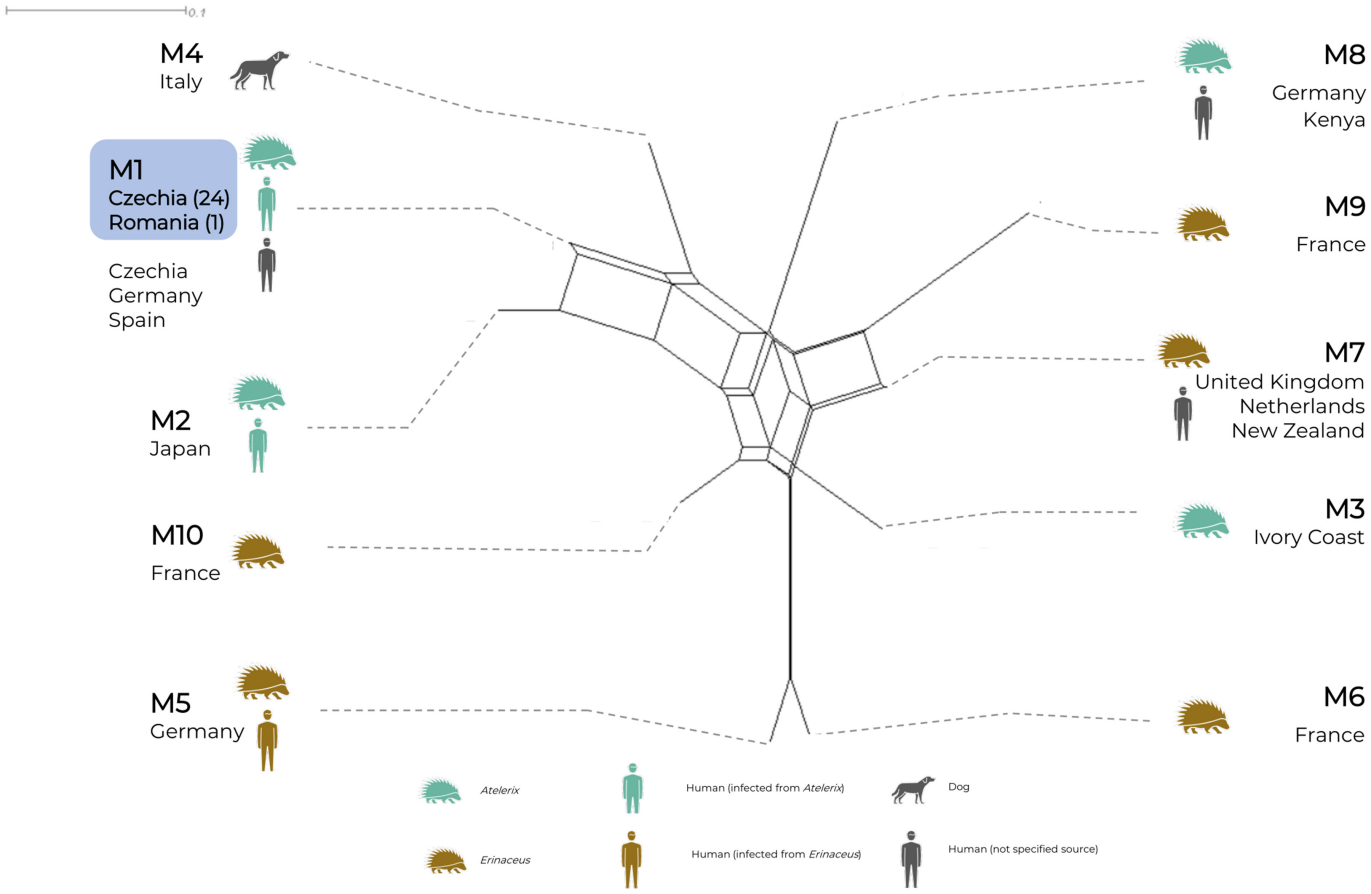
Multilocus microsatellite typing (MLMT) of *Trichophyton erinacei* isolates of pet *A. albiventris* from the Czech Republic and Romania. All 25 isolates belonged to the same microsatellite genotype, designated as the M1 genotype (2), defined by the number of repeats at each locus: CT21 = 4, CT21b = 16, TAG16 = 10, TC20 = 10, TCA16 = 6, TC19 = 5 and TC17a = 16.

The confirmed *T. erinacei* isolates were subjected to MLMT. Based on MLMT using seven microsatellite loci, all strains were assigned to the M1 genotype (Figure 3).

## 4 Discussion

*Trichophyton erinacei*, along with *T. benhamiae* and *T. quinckeanum*, is among the zoonotic dermatophytes frequently designated as emerging pathogens due to their increasing incidence in recent years (6, 10, 28, 39–42). Importantly, and not surprisingly, these *Trichophyton* species are associated with pet animals. Hedgehogs (a primary source of *T. erinacei*), guinea pigs (*T. benhamiae*), and rodents/cats (*T. quinckeanum*) are frequently handled by children, including in schools, petting zoos and similar facilities (6, 39, 40, 42). Given the high proportion of asymptomatic carriers among animals, timely and accurate diagnosis, along with appropriate antifungal treatment are essential for effective control and prevention of zoonotic transmission (17, 20).

In animal practice, collection of dermatophytes typically relies on non-invasive or semi-invasive techniques. However, this can still be challenging in species like hedgehogs, whose natural defense mechanism of curling up into a ball makes effective examination and sampling more difficult. In some cases, safe and effective sample collection may require immobilization and anesthesia, which carries risks and adds costs, especially under field conditions or when performed by untrained personnel (43, 44).

Inspired by previous studies using non-invasive sampling for dermatophytes (5, 31–34), we aimed to identify the most effective sampling method for detecting *T. erinacei* in pet hedgehogs using simple and widely available tools. Recognizing the need for efficient and widely applicable sampling strategy, we tested materials that are both inexpensive and readily available.

Among the three different sampling techniques, swabbing the skin surface with interdental brushes proved to be a most sensitive in detecting *T. erinacei*, compared to cotton swabs and toothbrushes. Notably, negative results from all three swabbing techniques did not always indicate true absence of the pathogen. In two animals, *T. erinacei* was detected only from additional material (spines or crusts), despite all swabs testing negative. Of all the possible combinations of the three techniques, using an interdental brush and a toothbrush together is the most effective. However, animal welfare must be considered, as using a toothbrush may cause a stronger stress response.

Comparative studies evaluating the effectiveness of swabbing techniques have been conducted in both asymptomatic children (45) and cats (34) to detect dermatophyte carriage. A total of 21 asymptomatic carriers (1.3%) were detected from 1592 children in Turkey, the hairbrush was found to be the most effective (64%), followed by the cotton swab (20%), and toothbrush (16%) (45). Among cats positive for *Microsporum canis* (*n* = 39), 23% (9/39) were asymptomatic carriers and both, toothbrushes and carpet squares yielded comparable detection rates (34). These findings suggest that refining or combining sampling materials and methods could improve the detection accuracy of dermatophytes across different hosts, ultimately supporting more reliable diagnosis and targeted treatment strategies.

The inclusion of additional material (spines, scales, or crusts) in the diagnostic work-up increased diagnostic sensitivity. Additional material was collected from six hedgehogs with skin lesions (a subset of 13 animals with visible lesions), and scraping of the lesion was performed with the owner's consent. Cultures were positive in five of the six cases. Notably, in two of these animals, *T. erinacei* was detected exclusively from the additional material, while all corresponding swabs yielded negative results. This highlights that relying solely on swabs may underestimate the prevalence of dermatophytosis and that incorporating lesional material into routine sampling protocols may further enhance diagnostic sensitivity.

During sampling, interdental brushes and toothbrushes were generally well tolerated, while cotton swabs often triggered a stress response—tendency to coil. In several cases, spines detached spontaneously during handling. Based on these findings, using an interdental brush combined with collection of lesional material (such as hairs, scales, and spines) appears to be the most practical and effective diagnostic approach for symptomatic animals.

A possible explanation for the higher positivity rate observed with the interdental brush sampling method may lie in its structural characteristics and sampling efficiency. The elongated shape and compact arrangement of the bristles likely facilitate closer contact with the skin surface between the spines and hairs, thereby improving the collection of skin scales and hairs containing fungal material. In addition, the shorter bristles, compared with those of a regular toothbrush, may allow more effective transfer of the collected material onto the agar surface during inoculation, which could have contributed to the higher detection rate of *T. erinacei* observed with this method.

In our study, 24.3% (25/103) of the sampled hedgehogs tested positive, with most of these individuals being asymptomatic (76%; 19/25). Comparable data from other sources are relatively limited. Notably, our results differ from those reported in a Spanish study by Abarca et al. (1), which documented a 50% positivity rate (10/20)—including nine *A. albiventris* and one *H. auritus*—and an asymptomatic carrier rate of only 10% (1/10), this individual was kept together with positive hedgehog with skin changes. However, the Spanish study exclusively targeted hedgehogs with suspected dermatophytosis, which likely contributed to the higher observed prevalence and almost no asymptomatic carriers. A study from Japan reported a prevalence of 39% positive individuals (7/18) among pet hedgehogs, although no information was provided about asymptomatic carriers (46). Our study is the first to provide comprehensive data on the prevalence of *T. erinacei* in pet hedgehogs, including information on the occurrence of asymptomatic carriers.

Among wild *A. albiventris*, a study from Kenya found a prevalence of 22.2% (10/45), with 60% of positive animals being asymptomatic (29). Similarly, research from the Ivory Coast reported that 5 out of 8 hedgehogs were positive under microscopic examination, while fungal isolates were successfully obtained from 3 animals (30). In both African studies, the isolates were identified as *Arthroderma benhamiae*. However, Padhye and Ajello (47) and Takahashi et al. (46) later revealed that the isolates are *T. erinacei*, a finding further supported by Cmoková et al. (2).

More information about prevalences of *T. erinacei* in hedgehogs is in previous studies of wild European hedgehogs—Great Britain 20%−25% (48), France 24% (5), New Zealand 44.7% (7).

Our findings confirm the risk of infection by *T. erinacei* for workers in pet shops and contact zoo workers and visitors, hedgehog owners and veterinary staff (6, 14, 49).

A limitation of the present study is the absence of direct microscopic examination of the original samples. Direct microscopy is a valuable diagnostic tool in mycology, enabling rapid detection and visualization of the fungal elements directly in clinical material. However, in this study, samples were collected as part of a population-level screening approach, including both asymptomatic and symptomatic animals, without prior knowledge of fungal presence or lesion localization. Under these conditions, the application of direct microscopy, particularly in asymptomatic animals, was not feasible and would likely have had limited diagnostic yield. Future studies focusing on clinically affected animals could benefit from incorporating direct microscopic examinations to complement culture and molecular methods.

The finding of genetically identical *T. erinacei* strains in all analyzed pet *A. albiventris* specimens suggest a shared origin and clonal spread of the pathogen. This corroborates the findings of Cmoková et al. (2), who demonstrated that the *T. erinacei* has diverged into two subpopulations: one found in wild *Erinaceus* hedgehogs with a relatively high genetic diversity and the other in pet *Atelerix* hedgehogs with a low genetic variability and signs clonal transmission.

Previous studies have shown that *T. erinacei* occurs in the wild mostly in hedgehogs of the genus *Erinaceus* (2, 5–7, 48). In contrast, findings from wild *Atelerix* hedgehogs are extremely rare, with only few historical strains molecularly characterized and isolated in Kenya and Ivory Coast in the 1970s (2, 29, 30). The precise origin of the *T. erinacei* subpopulation associated with pet hedgehogs remains unclear. The genetic uniformity of *T. erinacei* strains in captive hedgehogs suggests a common origin of the infection. The prevalence patterns observed in our study suggest a single or limited introduction event followed by widespread distribution (50, 51).

## Data Availability

The original contributions presented in the study are included in the article/supplementary material, further inquiries can be directed to the corresponding author.
