# Supplementary material for: Zoonotic dermatophyte Trichophyton erinacei in pet hedgehogs: sampling methodology and further insight into genetic diversity
Source: Front Vet Sci. 2026 Jul 1;13:1755936. doi: 10.3389/fvets.2026.1755936 (PMC13368544; doi:10.3389/fvets.2026.1755936)
Supplement: Supplementary file 1 [file Data_Sheet_1.PDF]

Supplementary Table 1: The table provides supplementary information describing each sample in terms of species, locality, date of collection, sex, age, weight, origin and clinical signs. In addition, it includes data on the performance of the tested techniques, specifying their detection rate for each sample. The table also lists the reference standard used for comparison, allowing the reliability and accuracy of the tested methods to be evaluated relative to an established benchmark.

Code	Species	Interdental brush	Cotton swab	Toothbrush	Other material	Reference standard Trichophyton erinacei	Accession numbers ITS	Accession numbers LSU	Country	Locality	Year	Month	Sex	Age (m)	Weight (g)	Origin/source	Clinical signs
AAV1	*Atelerix albiventris*	positive	negative	negative	positive	positive	PX898672	PX898706	Czech Republic	Prague	2023	February	M	5	295	private breeder	dermatitis
AAV2	*Atelerix albiventris*	positive	positive	positive	positive	positive	PX898673	PX898707	Czech Republic	Prague	2023	February	M	5	343	private breeder	dermatitis
AAV3	*Atelerix albiventris*	positive	negative	negative	X	positive	PX898674	PX898708	Czech Republic	Prague	2023	February	M	5	330	private breeder	
AAV4	*Atelerix albiventris*	positive	negative	positive	X	positive	PX898675	PX898709	Czech Republic	Prague	2023	February	M	8	441	private breeder	
AAV5	*Atelerix albiventris*	positive	positive	negative	positive	positive	PX898676	PX898710	Czech Republic	Prague	2023	February	M	8	441	private breeder	dermatitis
AAV6	*Atelerix albiventris*	positive	positive	negative	X	positive	PX898677	PX898711	Czech Republic	Prague	2023	February	M	5	250	private breeder	
AAV7	*Atelerix albiventris*	positive	positive	positive	X	positive	PX898678	PX898712	Czech Republic	Prague	2023	February	M	5	250	private breeder	
AAV8	*Atelerix albiventris*	positive	negative	negative	X	positive	PX898679	PX898713	Czech Republic	Prague	2023	February	M	5	250	private breeder	
AAV9	*Atelerix albiventris*	negative	positive	negative	X	positive	PX898680	PX898714	Czech Republic	Prague	2023	February	M	5	250	private breeder	
AAV10	*Atelerix albiventris*	negative	positive	positive	X	positive	PX898681	PX898715	Czech Republic	Prague	2023	February	M	5	250	private breeder	WHS
AAV11	*Atelerix albiventris*	positive	positive	positive	X	positive	PX898682	PX898716	Czech Republic	Prague	2023	February	M	5	250	private breeder	tumor, dermatitis
AAV12	*Atelerix albiventris*	positive	negative	negative	X	positive	PX898683	PX898717	Czech Republic	Prague	2023	February	M	7	434	private breeder	
AAV13	*Atelerix albiventris*	negative	negative	negative	X	negative			Czech Republic	Prague	2023	February	M	5	369	private breeder	
AAV14	*Atelerix albiventris*	positive	negative	negative	X	positive	PX898684	PX898718	Czech Republic	Prague	2023	February	M	5	379	private breeder	
AAV15	*Atelerix albiventris*	negative	negative	negative	X	negative			Czech Republic	Prague	2023	February	M	5	342	private breeder	
AAV16	*Atelerix albiventris*	positive	negative	negative	X	positive	PX898685	PX898719	Czech Republic	Prague	2023	February	M	5	347	private breeder	
AAV17	*Atelerix albiventris*	positive	negative	negative	X	positive	PX898686	PX898720	Czech Republic	Prague	2023	February	M	8	532	private breeder	
AAV18	*Atelerix albiventris*	positive	negative	negative	X	positive	PX898687	PX898721	Czech Republic	Prague	2023	February	M	5	353	private breeder	
AAV19	*Atelerix albiventris*	positive	negative	negative	X	positive	PX898688	PX898722	Czech Republic	Prague	2023	February	F	5	362	private breeder	
AAV20	*Atelerix albiventris*	negative	negative	negative	X	negative			Czech Republic	Police n. M.	2023	June	M	5	250	private breeder	
AAV21	*Atelerix albiventris*	negative	negative	negative	X	negative			Czech Republic	Police n. M.	2023	June	F	5	250	private breeder	
AAV22	*Atelerix albiventris*	positive	negative	negative	X	positive	PX898689	PX898723	Czech Republic	Police n. M.	2023	June	F	5	250	private breeder	
AAV23	*Atelerix albiventris*	negative	negative	negative	X	negative			Czech Republic	Police n. M.	2023	June	M	5	250	private breeder	
AAV24	*Atelerix albiventris*	negative	negative	negative	positive	positive	PX898690	PX898724	Czech Republic	Police n. M.	2023	June	F	5	250	private breeder	
AAV25	*Atelerix albiventris*	positive	negative	positive	X	positive	PX898691	PX898725	Czech Republic	Police n. M.	2023	June	F	36	350	private breeder	dermatitis (spines, ears)

AAV26	*Atelerix albiventris*	positive	positive	positive	X	positive	PX898692	PX898726	Czech Republic	Police n. M.	2023	June	F	9	270	private breeder	
AAV27	*Atelerix albiventris*	positive	negative	positive	X	positive	PX898693	PX898727	Czech Republic	Police n. M.	2023	June	M	4	250	private breeder	
AAV28	*Atelerix albiventris*	negative	negative	negative	X	negative			Czech Republic	Police n. M.	2023	June	F	4	250	private breeder	
AAV29	*Atelerix albiventris*	negative	negative	negative	X	negative			Czech Republic	Police n. M.	2023	June	F	4	250	private breeder	
AAV30	*Atelerix albiventris*	negative	negative	negative	X	negative			Czech Republic	Police n. M.	2023	June	F	4	250	private breeder	
AAV31	*Atelerix albiventris*	negative	negative	negative	X	negative			Czech Republic	Police n. M.	2023	June	F	4	250	private breeder	
AAV32	*Atelerix albiventris*	negative	negative	negative	X	negative			Czech Republic	Police n. M.	2023	June	M	4	250	private breeder	
AAV33	*Atelerix albiventris*	negative	positive	negative	X	positive	PX898694	PX898728	Czech Republic	Police n. M.	2023	June	M	4	250	private breeder	
AAV34	*Atelerix albiventris*	negative	negative	negative	X	negative			Czech Republic	Police n. M.	2023	June	F	4	250	private breeder	
AAV35	*Atelerix albiventris*	negative	negative	negative	X	negative			Czech Republic	Police n. M.	2023	June	M	4	250	private breeder	
AAV36	*Atelerix albiventris*	negative	negative	negative	X	negative			Czech Republic	Police n. M.	2023	June	F	4	250	private breeder	
AAV37	*Atelerix albiventris*	negative	negative	negative	X	negative			Czech Republic	Police n. M.	2023	June	F	4	250	private breeder	
AAV38	*Atelerix albiventris*	negative	negative	negative	X	negative			Czech Republic	Police n. M.	2023	June	F	4	250	private breeder	
AAV39	*Atelerix albiventris*	negative	negative	negative	X	negative			Czech Republic	Police n. M.	2023	June	F	4	250	private breeder	
AAV40	*Atelerix albiventris*	negative	negative	negative	X	negative			Czech Republic	Police n. M.	2023	June	F	4	250	private breeder	
AAV41	*Atelerix albiventris*	negative	negative	negative	X	negative			Czech Republic	Police n. M.	2023	June	M	4	250	private breeder	
AAV42	*Atelerix albiventris*	negative	negative	negative	X	negative			Czech Republic	Police n. M.	2023	June	M	4	250	private breeder	
AAV43	*Atelerix albiventris*	negative	negative	negative	X	negative			Czech Republic	Police n. M.	2023	June	M	4	250	private breeder	
AAV44	*Atelerix albiventris*	negative	negative	negative	X	negative			Czech Republic	Police n. M.	2023	June	F	4	250	private breeder	
AAV45	*Atelerix albiventris*	negative	negative	negative	X	negative			Czech Republic	Police n. M.	2023	June	F	4	250	private breeder	
AAV46	*Atelerix albiventris*	negative	negative	negative	X	negative			Czech Republic	Police n. M.	2023	June	M	4	250	private breeder	
AAV47	*Atelerix albiventris*	negative	negative	negative	X	negative			Czech Republic	Police n. M.	2023	June	M	4	250	private breeder	
AAV48	*Atelerix albiventris*	negative	negative	negative	X	negative			Czech Republic	Police n. M.	2023	June	F	4	250	private breeder	
AAV49	*Atelerix albiventris*	negative	negative	negative	X	negative			Czech Republic	Police n. M.	2023	June	F	4	250	private breeder	
AAV50	*Atelerix albiventris*	negative	negative	negative	X	negative			Czech Republic	Prague	2023	September	M	15	500	private breeder	
AAV51	*Atelerix albiventris*	negative	negative	negative	X	negative			Czech Republic	Prague	2023	September	F	12	380	private breeder	
AAV52	*Atelerix albiventris*	negative	negative	negative	X	negative			Czech Republic	Prague	2023	September	M	18	360	private breeder	
AAV53	*Atelerix albiventris*	negative	negative	negative	negative	negative			Czech Republic	Prague	2023	September	F	5	360	private breeder	
AAV54	*Atelerix albiventris*	negative	negative	negative	X	negative			Czech Republic	Žalhostice	2023	September	F	6	340	private breeder	
AAV55	*Atelerix albiventris*	negative	negative	negative	X	negative			Czech Republic	Žalhostice	2023	September	F	8	440	private breeder	
AAV56	*Atelerix albiventris*	negative	negative	negative	X	negative			Czech Republic	Žalhostice	2023	September	F	36	600	private breeder	

AAV57	*Atelerix albiventris*	negative	negative	negative	X	negative			Czech Republic	Žalhostice	2023	September	F	36	550	private breeder	
AAV58	*Atelerix albiventris*	negative	negative	negative	X	negative			Czech Republic	Žalhostice	2023	September	M	18	N/A	private breeder	
AAV59	*Atelerix albiventris*	negative	negative	negative	X	negative			Czech Republic	Žalhostice	2023	September	F	36	600	private breeder	
AAV60	*Atelerix albiventris*	negative	negative	negative	X	negative			Czech Republic	Žalhostice	2023	September	M	15	318	private breeder	
AAV61	*Atelerix albiventris*	negative	negative	negative	X	negative			Czech Republic	Žalhostice	2023	September	F	36	520	private breeder	
AAV62	*Atelerix albiventris*	negative	negative	negative	X	negative			Czech Republic	Žalhostice	2023	September	M	30	518	private breeder	
AAV63	*Atelerix albiventris*	negative	negative	negative	X	negative			Czech Republic	Žalhostice	2023	September	F	7	450	private breeder	
AAV64	*Atelerix albiventris*	negative	negative	negative	X	negative			Czech Republic	Žalhostice	2023	September	F	6	350	private breeder	
AAV65	*Atelerix albiventris*	negative	negative	negative	X	negative			Czech Republic	Žalhostice	2023	September	F	6	450	private breeder	dermatitis (head, front limbs)
AAV66	*Atelerix albiventris*	negative	negative	negative	X	negative			Czech Republic	Žalhostice	2023	September	F	14	368	private breeder	
AAV67	*Atelerix albiventris*	negative	negative	negative	X	negative			Czech Republic	Žalhostice	2023	September	M	9	420	private breeder	
AAV68	*Atelerix albiventris*	negative	negative	negative	X	negative			Czech Republic	Žalhostice	2023	September	F	18	360	private breeder	
AAV69	*Atelerix albiventris*	negative	negative	negative	X	negative			Czech Republic	Žalhostice	2023	September	M	24	410	private breeder	
AAV70	*Atelerix albiventris*	negative	negative	negative	X	negative			Czech Republic	Žalhostice	2023	September	F	7	400	private breeder	
AAV71	*Atelerix albiventris*	negative	negative	negative	X	negative			Czech Republic	Žalhostice	2023	September	F	12	375	private breeder	
AAV72	*Atelerix albiventris*	negative	negative	negative	X	negative			Czech Republic	Žalhostice	2023	September	F	6	350	private breeder	
AAV73	*Atelerix albiventris*	negative	negative	negative	X	negative			Czech Republic	Žalhostice	2023	September	M	12	400	private breeder	
AAV74	*Atelerix albiventris*	negative	negative	negative	X	negative			Czech Republic	Žalhostice	2023	September	F	24	460	private breeder	
AAV75	*Atelerix albiventris*	negative	negative	negative	X	negative			Czech Republic	Žalhostice	2023	September	F	12	650	private breeder	
AAV76	*Atelerix albiventris*	negative	negative	negative	X	negative			Czech Republic	Žalhostice	2023	September	F	18	420	private breeder	
AAV77	*Atelerix albiventris*	negative	negative	negative	X	negative			Czech Republic	Žalhostice	2023	September	F	8	410	private breeder	
AAV78	*Atelerix albiventris*	negative	negative	negative	X	negative			Czech Republic	Žalhostice	2023	September	F	12	378	private breeder	
AAV79	*Atelerix albiventris*	negative	negative	negative	X	negative			Czech Republic	Žalhostice	2023	September	F	6	362	private breeder	
AAV80	*Atelerix albiventris*	negative	negative	negative	X	negative			Czech Republic	Žalhostice	2023	September	M	36	330	private breeder	
AAV81	*Atelerix albiventris*	negative	negative	negative	X	negative			Czech Republic	Žalhostice	2023	September	M	24	358	private breeder	
AAV82	*Atelerix albiventris*	negative	negative	negative	X	negative			Czech Republic	Žalhostice	2023	September	M	12	370	private breeder	
AAV83	*Atelerix albiventris*	negative	negative	negative	X	negative			Czech Republic	Žalhostice	2023	September	F	17	470	private breeder	
AAV84	*Atelerix albiventris*	negative	negative	negative	X	negative			Czech Republic	Chomutov	2023	November	F	30	370	private breeder	
AAV85	*Atelerix albiventris*	negative	negative	negative	X	negative			Czech Republic	Chomutov	2023	November	F	30	370	private breeder	
AAV86	*Atelerix albiventris*	negative	negative	negative	X	negative			Czech Republic	Prague	2023	December	F	18	600	private breeder	dermatitis (spines)
AAV87	*Atelerix albiventris*	negative	negative	negative	X	negative			Czech Republic	Prague	2023	December	M	60	300	private breeder	dermatitis (spines, ears, limbs)

AAV88	*Atelerix albiventris*	negative	negative	negative	X	negative			Czech Republic	Prague	2023	December	M	36	462	private breeder	dermatitis (spines, ears)
AAV89	*Atelerix albiventris*	negative	negative	negative	X	negative			Czech Republic	Prague	2023	December	M		260	private breeder	
AAV90	*Atelerix albiventris*	negative	negative	negative	X	negative			Czech Republic	Prague	2023	December	M	36	350	private breeder	dermatitis (spines)
AAV91	*Atelerix albiventris*	positive	negative	positive	X	positive	PX898695	PX898729	Czech Republic	Prague	2023	December	M	N/A	276	private breeder	dermatitis (head, ears)
AAV92	*Atelerix albiventris*	negative	negative	negative	X	negative			Czech Republic	Prague	2023	December	M	N/A	N/A	private breeder	
AAV93	*Atelerix albiventris*	negative	negative	negative	X	negative			Czech Republic	Prague	2023	December	M	N/A	400	private breeder	
AAV94	Atelerix albiventris	negative	negative	negative	X	negative			Czech Republic	Prague	2023	December	F	30	495	private breeder	
AAV95	Atelerix albiventris	negative	negative	negative	X	negative			Czech Republic	Prague	2023	December	F	72	350	private breeder	
AAV96	Atelerix albiventris	negative	negative	negative	X	negative			Czech Republic	Prague	2023	December	M	24	360	private breeder	
AAV97	Atelerix albiventris	negative	negative	negative	X	negative			Czech Republic	Prague	2023	December	M	N/A	600	private breeder	dermatitis (spines)
AAV98	Atelerix albiventris	negative	negative	negative	X	negative			Czech Republic	Prague	2023	December	F	48	300	private breeder	dermatitis (head, hind limbs)
AAV99	Atelerix albiventris	negative	negative	negative	X	negative			Czech Republic	Prague	2023	December	M	24	N/A	private breeder	
AAV100	Atelerix albiventris	negative	negative	negative	X	negative			Czech Republic	Prague	2023	December	F	36	410	private breeder	
AAV101	Atelerix albiventris	negative	negative	negative	X	negative			Romania	Cluj-Napoca	2023	December	M	12	265	petshop	
AAV102	Atelerix albiventris	negative	negative	negative	X	negative			Romania	Cluj-Napoca	2023	December	F	36	415	petshop	
AAV103	Atelerix albiventris	negative	negative	negative	positive	positive	PX898696	PX898730	Romania	Cluj-Napoca	2023	December	F	48	360	petshop	
